# Non-invasive imaging and cellular tracking of pulmonary emboli by near-infrared fluorescence and positron-emission tomography

**DOI:** 10.1038/ncomms9448

**Published:** 2015-10-01

**Authors:** Michael J. Page, André L. Lourenço, Tovo David, Aaron M. LeBeau, Fiore Cattaruzza, Helena C. Castro, Henry F. VanBrocklin, Shaun R. Coughlin, Charles S. Craik

**Affiliations:** 1 Department of Pharmaceutical Chemistry, University of California, San Francisco, California 94158-2517, USA; 2 CAPES Foundation, Ministry of Education of Brazil, Brasília DF 70040-020, Brazil; 3 LABiEMol, Postgraduate Program in Pathology, Universidade Federal Fluminense, Niterói, Rio de Janeiro RJ 23230-060, Brazil; 4 Cardiovascular Research Institute, University of California, San Francisco, California 94158-9001, USA; 5 Department of Radiology and Biomedical Imaging, University of California, San Francisco, California 94107, USA

## Abstract

Functional imaging of proteolytic activity is an emerging strategy to quantify disease and response to therapy at the molecular level. We present a new peptide-based imaging probe technology that advances these goals by exploiting enzymatic activity to deposit probes labelled with near-infrared (NIR) fluorophores or radioisotopes in cell membranes of disease-associated proteolysis. This strategy allows for non-invasive detection of protease activity *in vivo* and *ex vivo* by tracking deposited probes in tissues. We demonstrate non-invasive detection of thrombin generation in a murine model of pulmonary embolism using our protease-activated peptide probes in microscopic clots within the lungs with NIR fluorescence optical imaging and positron-emission tomography. Thrombin activity is imaged deep in tissue and tracked predominantly to platelets within the lumen of blood vessels. The modular design of our probes allows for facile investigation of other proteases, and their contributions to disease by tailoring the protease activation and cell-binding elements.

## Abstract (web summary)

Functional imaging of proteolytic activity is an emerging strategy to guide patient diagnosis and monitor clinical outcome. Here the authors present a peptide-based probe to detect and localize thrombin activity *ex vivo* and non-invasively in mouse models of wounding and pulmonary thrombosis.

## Graphical abstract

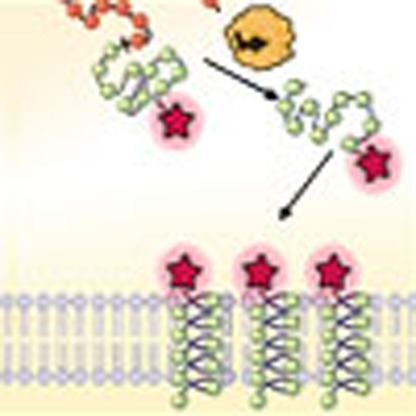

Functional imaging is an increasingly common approach to measure biological processes *in vivo* and offers the potential to improve patient diagnosis and clinical outcome^1^,^2^,^3^. Targeted imaging agents have traditionally relied on a one-to-one stoichiometric binding of probe to the target molecule for visualization. Enzyme-activated probes, developed to overcome this stoichiometric limitation, have typically suffered from low specificity and low signal intensity with high background noise^4^. Proteases are particular target enzymes of interest given their association with disease^5^. Pioneering work on coagulation factors^6^, cathepsins^7^ and matrix metalloproteases^8^ have found differential activity of these proteases throughout the progression of a number of diseases, including atherosclerosis^9^. Despite our greater understanding of proteases and their contribution to pathological conditions, practical issues have limited more widespread adoption of protease-based imaging in research and in the clinic. Thrombin—the pivotal protease of blood coagulation—exemplifies such issues^10^. Sensitive and specific thrombin probes are needed to better understand platelet and fibrinogen activation *in vivo*, which introduces factors that cannot be reproduced *in vitro* or *ex vivo*. In addition, such probes would be greatly beneficial in the detection and grading of clinically important diseases, such as myocardial infarction, stroke and pulmonary embolism^11^.

Past thrombin-targeted imaging agents have significant limitations emblematic of other protease-activated compounds. Long circulating half-lives and limited signal-to-noise ratios combine to hinder their utility with an invasive procedure often being required for probe detection. Previous designs also borrow heavily from historically successful *in vitro* approaches using synthetic peptide substrates whose specificity hinges on a few amino acid residues. These synthetic peptide substrates were created to assist in protein purification or to answer questions of mechanistic enzymology, and therefore, their *in vivo* applicability is limited^12^. Proteolytic turnover of short oligopeptide substrates is dominated by generic interactions in the protease active site, leading to considerable non-specific hydrolysis. Similar energetics underlie inhibitors of proteases, and inhibitor designs now aim to minimize contacts with the generic protease active site architecture to achieve selectivity that is meaningful *in vivo*. As proper registration within the active site is a prerequisite for catalysis^13^, we sought to create probes that could incorporate larger peptide targeting elements to maximize selectivity for individual proteases.

To build sensitivity for protease detection *in vivo*, we coupled cleavable substrates with probe-containing peptides that localize in cell membranes only after proteolytic release. We turned to peptides capable of spontaneous interaction with phospholipid membranes that prefer, but do not require, negatively charged surfaces as a scaffold. Often described as antimicrobial peptides (AMPs), with varying degrees of haemolytic activity, these polymers represent rich physicochemical diversity for agent modification^14^. We reasoned that such peptides would provide a subtle, yet established localization mechanism that likely could be influenced by proteolysis. AMP properties differ from previously described designs that incorporate highly hydrophobic groups^15^ or significant electrostatics for agent localization^16^,^17^,^18^, which can lead to non-specific accumulation. In particular, our agents aim to have limited non-specific interaction with healthy cells by possessing a hydrophilic imaging agent at one end and a protease-targeting sequence separated by a scissile bond on the other (Fig. 1a). Proteolysis facilitates agent interaction with phospholipid surfaces and allows insertion into the membrane interior by freeing one end of the peptide. As the concentration for detection is below the high copy number required to permeabilize membranes, the compounds have a good safety profile and do not affect hepatic or renal functions (Supplementary Fig. 4; Supplementary Table 2). We term these protease-responsive constructs restricted interaction peptides (RIPs) and found them capable of the non-invasive imaging of thrombin activity *in vivo* and also at monitoring this activity at the cellular level.

## Results

### Identification of RIPs

**Figure 1:**
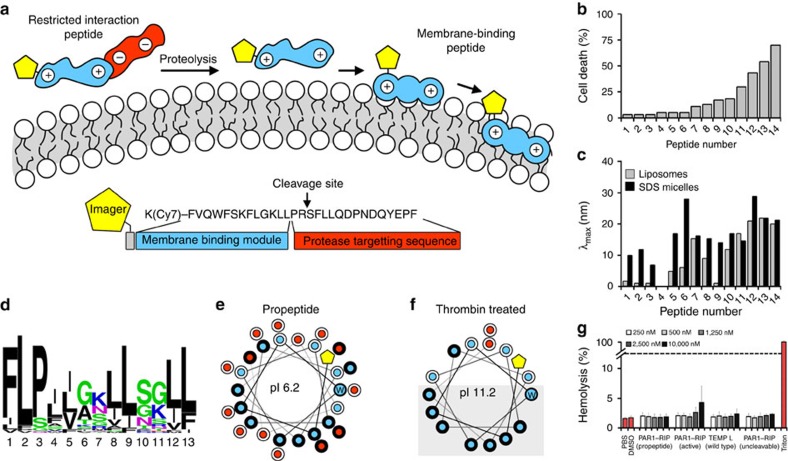
Mechanism underlying RIP technology. (**a**) RIP pro-peptides exist in a disordered state in solution with little affinity for phospholipid surfaces owing to the presence of the exosite-targeting sequence (red) and imaging agent (yellow) at the termini of the membrane-binding segment (blue). Following proteolysis, the membrane-binding segment is released and capable of interacting with cellular membranes whereupon they undergo conformational change and insertion. (**b**) Fourteen antimicrobial peptides (AMPs) were synthesized lacking their conventional post-translational modifications while including the Pro–Arg sequence that would be leftover after proteolysis by thrombin. The peptides were found to have various limited degrees of cellular toxicity. (**c**) Toxicity of these initial peptides weakly correlated with incorporation into PC liposomes or SDS micelles suggesting a variety of AMP mechanisms could be used in the RIP strategy and with different toxicities. (**d**) Temporin L (peptide 12 in (**b**) and (**c**)) in PAR1–RIP belongs to a large peptide family enabling its optimization when coupled to different imaging groups and targeting sequences. (**e**,**f**) Helical wheel diagram of the activated form of the peptide highlights the amphiphobic nature of the peptide. Colouring as in (**a**) with hydrophobic residues in black and hydrophilic residues in white. In contrast, the PAR1–RIP masks this helix with hydrophilic residues that also create an acidic pI for the peptide. (**g**) The resulting Cy7–PAR1–RIP probe selected for NIR imaging shows good haemocompatibility towards human erythrocytes in both active and pro-peptide form. Data are mean±s.d. of three biological replicates.

A library of potential membrane-interacting peptides, bearing the remnants of a protease cleavage site and absence of their natural post-translational modifications, was analysed to identify 14 peptides capable of binding phospholipid membranes post-proteolytic activation. Sequences were optimized to maintain the endogenous sequence of the AMP while maximizing the thrombin cleavage site (Fig. 1b–d; Supplementary Table 1). Temporin L (TempL) was chosen from this group on the basis of its relatively short sequence, large number of homologues, modest cationic disposition, conformational change into an α-helix on membrane binding and reasonable haemocompatibility to human blood cells (Fig. 1b,g)^19^,^20^. These factors enable facile synthesis, tunable potency, limited non-specific interactions, robust on and off state definition and the ability to insert into eukaryotic cell membranes. The protease-activated receptor-1 (PAR1) activation site, a natural substrate of thrombin, was chosen to incorporate into the initial design owing to its known high sensitivity to the protease. Extended interaction of this substrate outside of the protease active site is known to mediate efficient recognition by thrombin, yet this has not been incorporated into past imaging agents owing to its size^6^. The chosen cleavage site and extended interaction sequence from PAR1 encompasses P2–P14′ in Schechter and Berger^21^ nomenclature and more than 1,000 Å^2^ buried surface area in the enzyme substrate complex^22^. The buried area compares favourably to antibody–antigen complexes and highlights the sensitivity enabled by the RIP architecture. A peptide whose sequence is composed of TempL followed by the sequence of PAR1 was synthesized and is referred to as PAR1–RIP.

### Protease-dependent interactions of PAR1–RIP constructs

**Figure 2:**
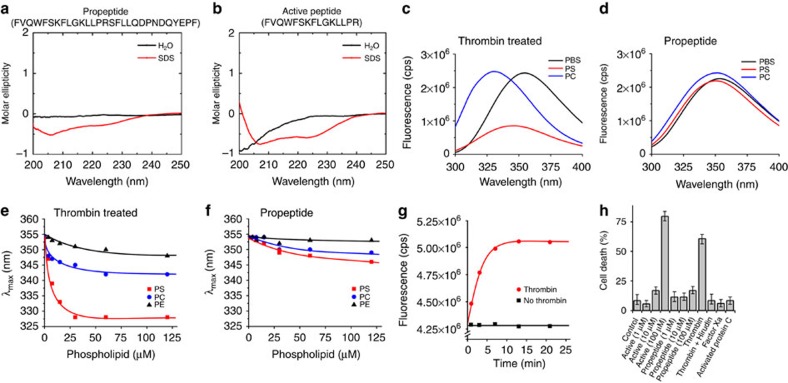
Biophysical and cellular confirmation of RIP technology lacking dye conjugates. (**a**–**d**) When incubated with SDS micelles or liposomes consisting of phosphatidylserine (PS) or phosphatidylcholine (PC) thrombin-treated PAR1–RIP inserts into each membrane evident by circular dichroism (**a**,**b**) and the decrease in maximum emission wavelength of its tryptophan within the membrane-binding segment. In contrast, the pro-peptide PAR1–RIP undergoes little change in spectra (**c**,**d**). (**e**) Titration of the activated peptide with increasing amount of liposomes reveals a preference for negatively charged PS membranes. (**f**) Slight changes in maximum emission wavelength and the near-linear response are indicative of limited interaction. (**g**) Co-incubation of thrombin (10 nM) with PAR1–RIP (8 μM) and PC liposomes (100 μM) demonstrates time-dependent proteolysis and indicated lack of non-specific association of the peptide. (**h**) Measuring cell viability using Trypan blue uptake reveals a clear difference in cellular effects of the pro-peptide, which is non-toxic, and its thrombin-activated form. Blocking proteolysis with the thrombin-specific inhibitor confirms the role of the protease. Data are mean±s.d. of three biological replicates. cps, counts per second.

PAR1–RIP has a slight anionic disposition with a predicted pI of 6.2 in its pro-peptide form. When cleaved by thrombin two fragments are produced; one with a pI value of 11.2 that binds to membranes and another with a pI of 3.5 that diffuses away (Fig. 1e,f). The protease-dependent activation mechanism involves two steps. In the first, electrostatics and hydrophilicity hinder the ability of the probe to associate with the phospholipid membrane and penetrate into the hydrophobic core. Second, amino acid residues in the target sequence and the properties of imaging agent hinder the peptide from forming an α-helix at the solvent membrane interface as observed in circular dichroism spectra (Fig. 2a,b). Proteolysis, therefore, aims to enable both membrane association and insertion of the previously restricted probe.

Measurement of changes in the intrinsic fluorescence of the singular tryptophan residue in the membrane-interacting segment demonstrated that the thrombin-cleaved form indeed shows markedly enhanced peptide insertion into lipid compared with the pro-form. A 10-nm decrease in the maximum emission wavelength of the thrombin-cleaved form indicated burial of this residue that resides in the middle of the membrane-binding module into the apolar phosphatidylcholine (PC) liposomes (Fig. 2c). Similar restriction and post-proteolysis binding was observed with both zwitterionic and anionic detergent micelles suggesting that high membrane curvature does not impact probe behaviour and the discrimination is robust (Supplementary Fig. 1). Association with the anionic phosphatidylserine (PS) liposomes was also evident by the decrease in signal intensity presumably reflecting proximity of the tryptophan to the negative charge. In contrast, the intact pro-peptide had limited interaction with PC phospholipid membranes evident by the <3-nm shift in the maximum emission wavelength of its spectral profile in the presence of liposomes consisting of either PC or PS and quenching was not observed (Fig. 2d). As anticipated from the use of an AMP with a cationic disposition in our design, we observed a preference for anionic lipids, weaker interaction with zwitterionic surfaces and little affinity for cationic membranes (Fig. 2e). As the maximum emission wavelength from the pro-peptide does not significantly decrease at high molar ratios of phospholipid, we infer that the peptide does not insert into the membrane and the observed signal reflects non-specific binding and a limit of the experiment (Fig. 2f). Indeed, when thrombin was directly added to mixtures containing the pro-peptide and PC liposomes, proteolysis of the substrate was observed indicating that the pro-peptide does not associate with the membrane and is available for proteolytic activation (Fig. 2g). Other peptide compositions such as TempL, coupled with the corresponding sequence of PAR2 (pI 10.6) bound constitutively to lipid evident by their maximum emission wavelength decrease in the presence of sodium dodecyl sulphate (SDS) micelles (Supplementary Fig. 2). These alternate designs indicate overall charge, position of charge and the potency of the membrane-binding segment contribute in different ways to the extent of restricted interaction with phospholipid surfaces and can be modified to tune probe properties.

Discrimination between on and off states in PAR1–RIP observed *in vitro* translated *in vivo*. To gauge the interaction of RIP probes with cells, we measured their toxicity at ∼6- to 600-fold excess of the intended use *in vivo*. Human Jurkat cells in suspension tolerated micromolar levels of dye-free PAR1–RIP pro-peptide. However, the purified active form of PAR1–RIP or co-incubation with thrombin, but not the proteases factor Xa (FXa) and activated protein C, mediated membrane-permeabilization and uptake of Trypan blue in the low micromolar range (Fig. 2h). Inclusion of the highly specific thrombin inhibitor hirudin blocked these protease-dependent effects and confirmed the key role for thrombin in this process. Cells grown on plastic were incubated with 100 μM pro-peptide to confirm that uptake of the fluorescent dye DRAQ7 only occurred in the protease-treated form (Supplementary Fig. 3a) not the pro-peptide alone (Supplementary Fig. 3b). Concentrations required to mediate toxicity in cell culture exceed those used in animal studies described below by several orders of magnitude, but provide a useful readout nonetheless. Protease-dependent membrane-binding properties with lipids and cells suggested PAR1–RIP fit our design criteria and would bind eukaryotic membranes after proteolytic activation with a preference for PS.

### Kinetics and properties of dye-conjugated PAR1–RIP

**Figure 3:**
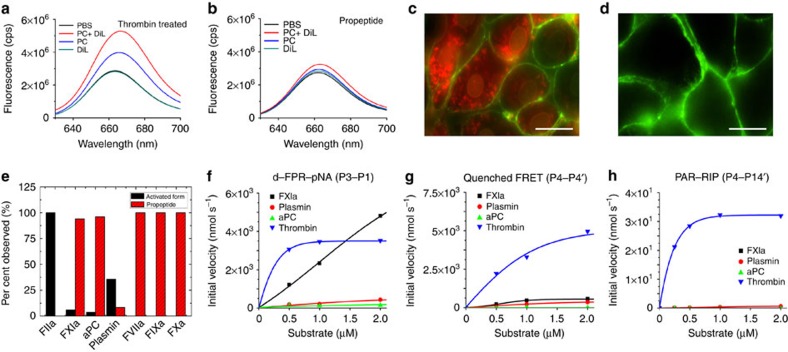
Biophysical and cellular confirmation of RIP technology bearing imaging conjugates. (**a**) Similar protease-dependent membrane interaction is observed when PAR1–RIP contains a fluorophore, such as Cy5, at its N terminus. Binding to liposomes that contain a donor fluorophore at equimolar concentrations such as the Cy3 equivalent 1-1′-dioctadecyl-3-3-3′-3′-tetramethylindocarbocyanine (DiL) is evident by FRET using the thrombin-activated Cy5–PAR1–RIP (100 nM peptide, 10 nM thrombin, 1 h). (**b**) In contrast, little FRET signal is apparent in the pro-peptide indicated that it does not associate with phospholipid membranes non-specifically. (**c**) Cy7–PAR1–RIP (red) exhibits protease-dependent interaction with cells in culture (scale bar, 10 μm) as its thrombin-activated form interacts with the membrane, is taken up, and redistributes to interior membranes over time. (**d**) The pro-peptide (0.5 μM) shows limited interaction with cells (scale bar, 10 μm). (**e**) Tandem HPLC/mass spectrometry analysis evaluates the proteolytic sensitivity of the probe to a broad spectrum of human proteases. (**f**) Conventional biochemical approaches to measure the kinetics of proteolysis use small chromogenic substrates such as the tripeptide d–Phe–Pro–Arg–paranitroanilide (pNA) that lack the power to discriminate closely related proteases. (**g**) Lengthening the substrate used improves specificity but noticeable turnover by other proteases still occurs and such probes are not amenable for *in vivo* application. (**h**) Inclusion of exosite-recognition in PAR1–RIP both decreases the *K*_*M*_ for recognition by the target protease thrombin and rate of activation by thrombin and more so for other closely related proteases resulting in a *k*_*cat*_*/K*_*M*_ of 8.4±0.3 × 10^6^ M^−1^ s^−1^, which is >100-fold higher than towards other proteases. Initial velocities are normalized to 10 nM of protease in each panel as the assays were performed at different concentrations ensuring linear response ranges over the first 5% of substrate hydrolysis.

PAR1–RIP bearing an N-terminal cysteine was labelled with a variety of fluorescent dyes (Supplementary Fig. 8) using maleimide chemistry and found to retain restricted membrane interaction that could be unleashed by selective proteolysis. Fluorescence resonance energy transfer (FRET) between the peptide and an appropriate lipophilic dye demonstrated probe association with phospholipid membranes in a protease-dependent fashion when carrying a fluorescent dye (Fig. 3a,b). In certain formulations, such as those containing Cy5, membrane binding of the active form was also evident by up to a 50% increase in fluorescence intensity suggesting the activated form of the peptide could drag the fluorophore, at least partly, into the apolar membrane interior and improve its optical characteristics. Successful RIP formulations included dyes with a zwitterionic or negatively charged nature. Fluorescein, tetramethylrhodamine and cyanine dyes (Cy3, Cy5 and Cy7) were found amenable, whereas, only certain ATTO series dyes like ATTO 680 demonstrated protease-dependent activity. ATTO 565 was not restricted when conjugated on the N terminus of the TempL–PAR1 peptide. This effect was attributed to the cationic and lipophilic nature of this dye, which allowed the peptide to sample the membrane interior and thus form a stable partially buried helix. These data suggest RIP technology is capable of carrying payloads of varying size, charge and complexity to membrane surfaces with limited caveats.

Protease-dependent cell interactions were observed with Cy7–PAR1–RIP, which bears a near-infrared (NIR) fluorophore later used for non-invasive animal imaging in studies described below (Fig. 3c,d). The pattern of cellular uptake of fluorophore-conjugated activated peptide, assessed by live-cell epifluorescence microscopy, followed a typical sequence involving surface labelling in minutes followed by endosomal uptake over the next hour with redistribution into intracellular membranes. Together these studies confirmed the restricted membrane interaction of PAR1–RIP and its selective activation by thrombin extends to cell-based phospholipids leading to probe deposition and internalization.

PAR1–RIP was selective for the target protease thrombin and exhibited an efficient Michaelis constant (*K*_*M*_) for recognition as previously observed with peptides derived from the PAR1 sequence (Fig. 3e)^23^,^24^. Comparison of the rates of protease-mediated hydrolysis of a tripeptide chromogenic substrate (P3–P1), quenched oligopeptide substrate (P4–P4′) and our PAR1–RIP probe (P4–P14′) emphasize the value of a larger surface area of interaction between enzyme and substrate in RIP probes. When substrates have limited potential contacts with the protease, and the majority of them are shared with homologous enzymes, their turnover is non-specific. For example, the chromogenic substrate d–Phe–Pro–Arg–paranitroanilide is not only an excellent substrate for thrombin but also for many other proteases (Fig. 3f). As substrate length increases to include four additional residues on the prime side of the scissile bond, specificity increases 10-fold (Fig. 3g). Inclusion of the 10 amino acids responsible for exosite binding to thrombin further increases the relative specificity eightfold and reduces the *K*_*M*_ to a physiologically relevant value of 0.2±0.1 μM (mean±s.d., *n*=3; Fig. 3h). The kinetic advantages gained on exosite inclusion are similar to those reported in other substrate designs suggesting the membrane-binding module does not negatively influence binding characteristics^25^. Catalytic efficiency of thrombin-mediated PAR1–RIP activation, *k*_*cat*_*/K*_*M*_ of 8.4±0.3 × 10^6^ M^−1^ s^−1^, is 100- to 900-fold greater than previous thrombin-targeted probes that can localize to sites of its activity for *in vivo* imaging entirely due to its lowered *K*_*M*_^6^,^26^,^27^. Notably, proteases included in Fig. 3e were chosen by their ability to cleave PAR1–RIP with any measurable rate. Other proteases tested such as factors IXa, Xa and XIa, activated protein C, plasmin and tissue-type plasminogen activator did not cleave PAR1–RIP after 24 h of co-incubation (Fig. 3e). In isolation these proteases do not cleave PAR1 yet readily cleave small chromogenic substrates that bind the active site alone^28^. Several of these proteases are thought to signal through PARs *in vivo* through assistance from a co-factor^29^. Together these data indicate PAR1–RIP presents physiologically relevant kinetic parameters for its activation by thrombin even in the presence of a membrane-binding module.

### Imaging arterial thrombosis in the mouse carotid artery

**Figure 4:**
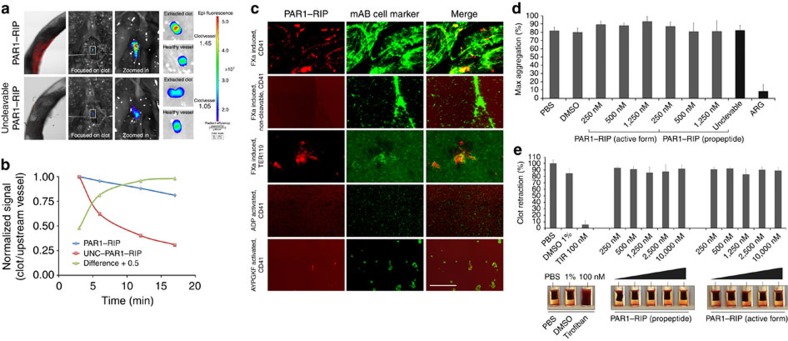
Probe deposition at sites of vascular injury as a function of thrombin generation. The generation of an occlusive clot is achieved by exposing the carotid artery of C57/B6 mice (*n*=3 per treatment group) with a Whatman disk soaked in FeCl_3_ solution (4%) for 3 min. Occlusion of the vessel is obtained as a function of thrombin generation and proteolytic activity within the injury. (**a**) The Cy5–PAR1–RIP probe accumulates in the injury site in a time-dependent manner, showing a distinct difference against its uncleavable variant (P1 alanine). After total occlusion (1 mm^3^ clot after 20 min), the clotted artery was removed and the fluorescence signal observed was compared with the adjacent healthy tissue of the artery. (**b**) Normalized signals from the clot/healthy vessel ratio reveals an increasing difference over time between PAR1–RIP and its uncleavable variant. These data corroborate to the importance of thrombin activity for probe turnover and localization at sites of high thrombogenicity. (**c**) Clotting of whole murine blood (scale bar, 100 μm) in a protease-dependent manner using coagulation FXa (50 nM) converts prothrombin to thrombin and causes fluorescein–PAR1–RIP (50 nM, red) to become activated and localize on platelets preferentially over red blood cells, which are indicated by the CD41 and TER119 antibody markers (green). Similar probe deposition is not observed with an uncleavable variant of the peptide or when the blood is stimulated by ADP or a PAR4-specific agonist (AYPGKF). (**d**) As observed by light transmission aggregometry (*n*=3), after incubation with PAR1–RIP at 37 °C in either pro-peptide and/or active form, platelet aggregation is not impaired by the probes, even at fivefold higher concentrations than that used for imaging. Late-stage platelet activation events, such as clot retraction (**e**) are also not altered by the probes, even at 40-fold higher concentrations than that used for imaging (*n*=3). Data are mean±s.d. of three biological replicates. All results are expressed as *P*<0.05, one-way ANOVA followed by Dunnett’s post-test.

Ferric chloride-induced endothelial damage in the carotid artery shows that Cy5–PAR1–RIP deposits intensely at the injury site in comparison to an adjacent healthy vessel. Under similar conditions of vascular damage an uncleavable version of the probe bearing an alanine at the P1 position accumulated with much less intensity at the injury site (Fig. 4a). The normalized signal of damaged/healthy vessel over time indicates that cleavage of the probe, rather than occlusion of the vessel, is the driving force behind PAR1–RIP deposition to the thrombotic lesion (Fig. 4b). Thus, PAR1–RIP probes accumulate at sites of thrombin proteolytic activity, even when no significant occlusion of the vessel can be observed.

### Imaging thrombin generation during *ex vivo* clot formation

PAR1–RIP labelled platelets during *ex vivo* blood clot formation (Fig. 4c). Murine whole blood was collected in the presence of sodium citrate and clotted by adding coagulation FXa and calcium in the presence of fluorescein–PAR1–RIP on glass slides. The pattern of probe accumulation was compared with fluorescent antibodies against CD41 (anti-mouse CD41 phycoerythrin, MWReg30 clone, eBioscience, 1:100 dilution), a marker of platelets, and TER119 (anti-mouse TER-119 phycoerythrin, TER-199 clone, eBioscience, 1:100 dilution) a marker of red blood cells. Probe accumulation overlapped that from CD41 yet was more heterogeneous in its location and signal intensity. Certain platelets within the clot showed little probe accumulation whereas others were intensely labelled. Under similar conditions of clot formation, an uncleavable variant of PAR1–RIP bearing an alanine at the P1 position accumulated with much less intensity on platelets. When whole murine blood was re-calcified and stimulated with platelet activators such as ADP or the AYPGKF peptide, PAR1–RIP neither accumulated on platelets nor other cell type indicating the insufficiency of P2Y1 and P2Y12 ADP receptors or PAR4 mediated platelet activation for probe accumulation and the need for thrombin to cleave the probe for a maximal response. Normal platelet functions, such as thrombin-induced aggregation (Fig. 4d) and clot retraction (Fig. 4e), were shown to be unaltered by concentrations of PAR1–RIP greater than those used in the imaging studies, × 5 and × 40, respectively. These observations highlight the responsiveness of PAR1–RIP to proteolysis and amplified by PS exposure, without interfering with normal haemostasis.

### Non-invasive detection of PE using NIR fluorescence

Preliminary analysis of biodistribution, pharmacokinetics and acute toxicology were assessed before inclusion of RIPs into standard clotting models. In mice, the small peptidic nature of the probe and absence of bulking agents led to a rapid clearance from the circulation and subsequent accumulation in the bladder that plateaus within 30 min (Supplementary Fig. 4). Three routes of clearance were evident—hepatic, renal and digestive. We therefore investigated whether PAR1–RIP had acute toxic effects on liver and kidneys and showed that most of the 21 measured serum biochemical parameters were not different from controls at all time points (Supplementary Table 2). Thus PAR1–RIP does not affect hepatic or renal functions and is not haemolytic at the tested dose. The rapid circulating half-life indicated that the probes would have, in the current formulation, applications for short-term experiments asking direct questions about thrombin generation. We therefore chose the thromboplastin-mediated pulmonary embolism model, which rapidly generates microscopic emboli in the lungs.

Pulmonary emboli (PE) could be detected and quantified non-invasively in a dose-dependent manner by PAR1–RIP conjugated to a variety of fluorescent and NIR dyes. Initial studies applied previously reported doses of thromboplastin at lethal levels and direct injection into the inferior vena cava of mice^30^ and showed ATTO 680–PAR1–RIP accumulated proportionally to the severity of the insult (Supplementary Fig. 5). We continued to use this PE model, but lowered the maximal dose of thromboplastin injected to 10-fold below the lethal level and turned to tail-vein injection as the route of administration to obviate signal due to surgical manipulation of the animal.

**Figure 5:**
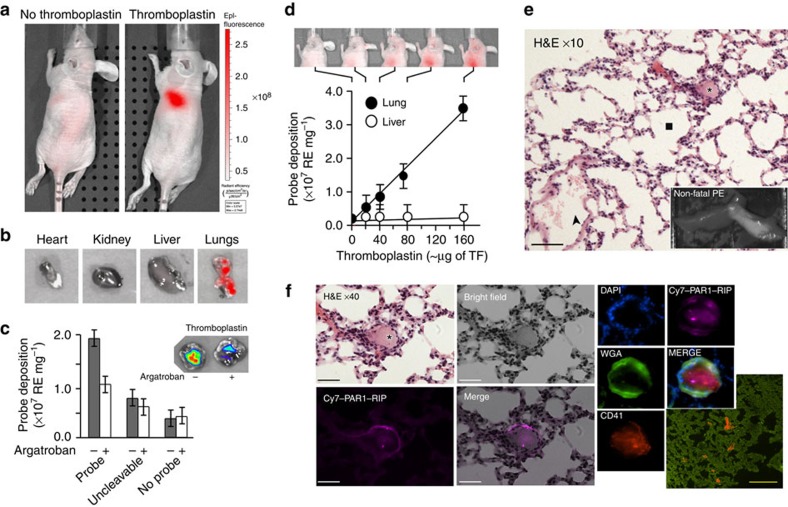
Non-invasive functional imaging of thrombin-mediated clots in a murine model of pulmonary embolism and its tracking to the cellular level. (**a**) Injection of Cy7–PAR1–RIP (500 pmol) with thromboplastin to *Foxn1*^*nu*^ mice (*n*=3 per treatment group) produces pulmonary emboli that can be detected non-invasively via near-infrared fluorescence within 15 min post injection. (**b**) Probe accumulates predominantly in the lungs in this model and not other organs. (**c**) Fluorescein-conjugated probe lung accumulation is correlated to proteolysis as an uncleavable variant accumulates significantly less and can be reduced by direct thrombin inhibitors like argatroban (indicated by − or +). (**d**) Non-invasive signal correlates with the amount of clotting agent and confirmed by *ex vivo* analysis of organ labelled normalized by its weight. The linear response curve reflects the use of non-lethal doses of thromboplastin more than 10-fold below the LD_50_. Data are mean±s.d. of three biological replicates. All results are expressed as *P*<0.05, one-way ANOVA followed by Dunnett’s post-test. (**e**) The Cy7 signal observed in non-invasive images and that of the extracted organs results from few microscopic emboli (
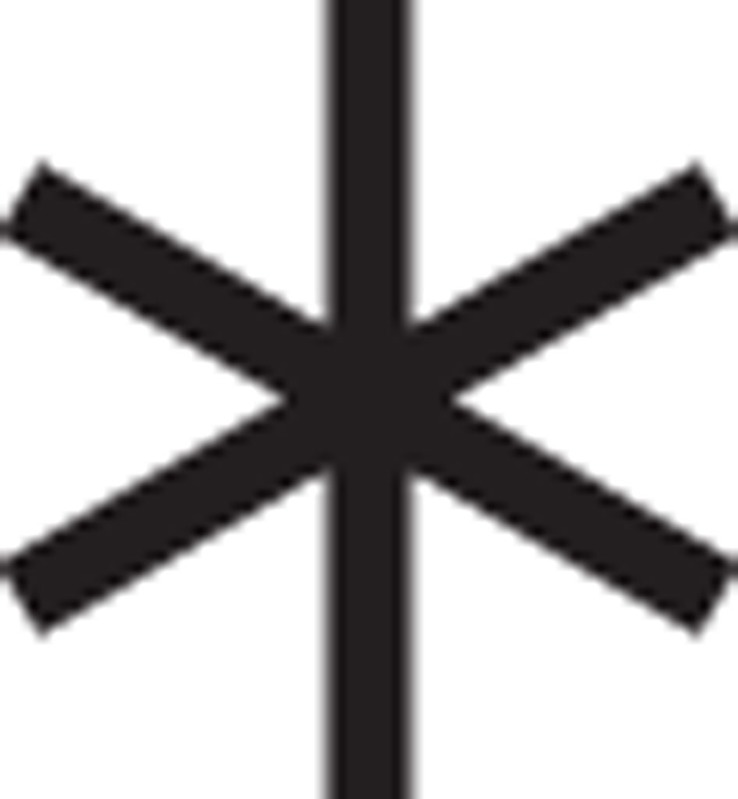
) generated through non-fatal doses of thromboplastin, whose fluorescence radiates from individual emboli rather than widespread probe deposition throughout the lung. Healthy pulmonary alveoli (▪) are preserved, as well as the (
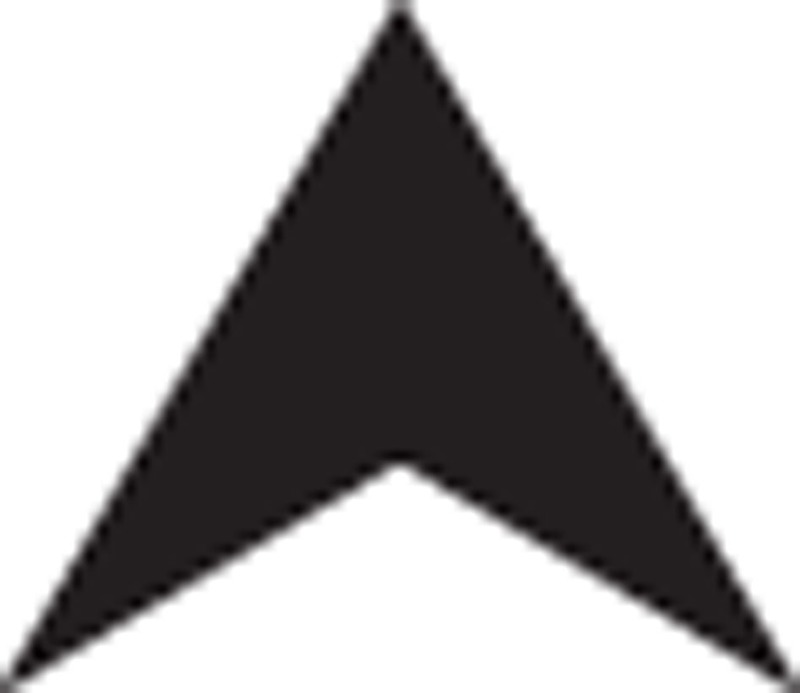
) circulation in bigger vessels (scale bar, 50 μm). (**f**) Cy7–PAR1–RIP (purple) accumulates within the lumen of the vessel and on or near platelets (CD41, red; scale bars, 25 μm white, 0.5 mm yellow). Cell nuclei revealed by DAPI (blue) and their membranes with the lipophilic dye WGA (green). RE, relative equilibrium; TF, tissue factor (thromboplastin).

Non-lethal PE were detected non-invasively within minutes of injection of picomolar amounts of Cy7–PAR1–RIP in mice bearing thromboplastin-induced emboli (Fig. 5a). Probe accumulated predominantly in the lungs, a direct outcome from the increased clotting activity promoted by thromboplastin in this pulmonary embolism model (Fig. 5b), and was excreted via renal and hepatic clearance. Using a non-cleavable version of fluorescein-conjugated PAR1–RIP and the thrombin-specific inhibitor argatroban, we observed reduction in probe accumulation near, but not equal to background levels indicating proteolysis of the probe was necessary, but not required, for its deposition in this model (Fig. 5c). The inability to completely abrogate accumulation presumably reflects both the non-specific accumulation of the probe within the clot as well as the inherent affinity of the uncleavable probe for thrombin. In contrast with ATTO 680-containing PAR1–RIP, the Cy7 variant exhibited more rapid *in vivo* clearance and presented markedly increased liver uptake. Nevertheless, lung deposition was quantitative and linear reflecting the low and non-lethal amount of thromboplastin injected (Fig. 5d). The intense signal, when viewed non-invasively or with extracted organs, resulted from Cy7–PAR1–RIP accumulation in a small number of intensely labelled emboli in the lung that could be tracked *ex vivo* (Fig. 5e,f), even in non-occluded vessels (Supplementary Fig. 6). The small number of emboli observed in the lung agrees with the linear response of probe accumulation as a function of thromboplastin, which is more than 10-fold below its reported LD_50_ (dose lethal to 50% of animals tested) in mice, because much of the lung tissue is normal and with unobstructed vessels at this dose of coagulant (Fig. 5e). The ability to track protease activity down to a precise cellular location highlights the broader utility of RIP technology beyond non-invasive detection. Signal from the Cy7 dye was detected throughout the emboli, but tended to co-localize with platelet-rich areas based on signal overlap with antiplatelet-CD41 and also haematoxylin and eosin staining (Fig. 5f; Supplementary Figs 6 and 7). These data indicate RIP technology can be used to couple non-invasive imaging of protease activity to its location at the cellular level.

### Real-time measurement of thrombin generation after wounding

**Figure 6:**
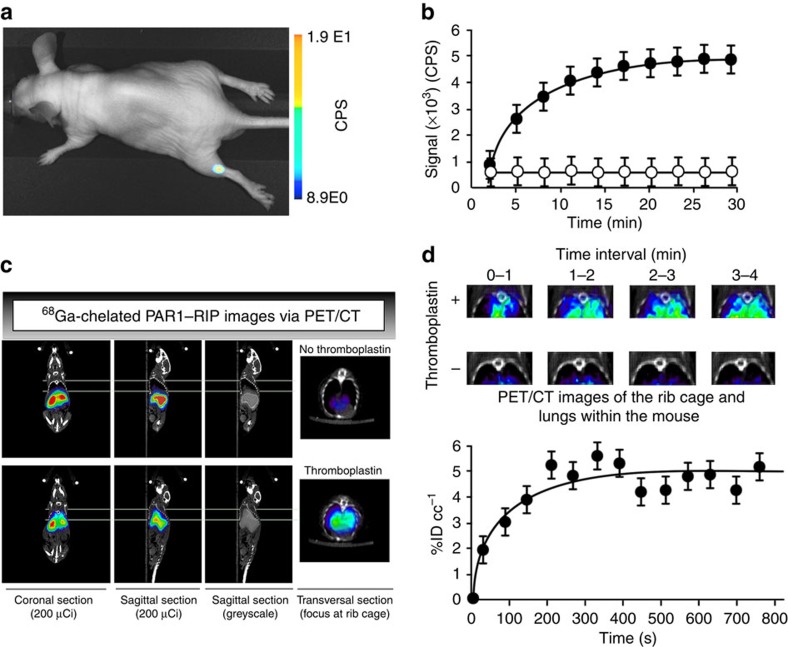
Real-time detection of thrombus generation using PAR1–RIP formulated for NIR fluorescence or PET imaging. (**a**) Puncture of the saphenous vein in mice (*n*=3 per group) creates a submillimeter-sized blood clot whose formation can be directly quantified (**b**) via a signal-to-noise increase of more than eightfold saturating in ∼20 min. Gain in signal is not due to background from the animal as the absence of the injury does not result in a detectable signal. (**c**) Non-lethal pulmonary emboli via tail-vein injection of thromboplastin were detected non-invasively by the clinically relevant imaging modality PET/CT using ^68^Ga-labelled DTPA–PAR1–RIP. Healthy mice (*n*=5 per treatment group) were tail-vein injected with 150–200 μCi of ^68^Ga–DTPA–PAR1–RIP and scanned dynamically for 20 min to detect thrombin generation in the lungs via PET. Accumulation in the liver is more pronounced using this variant of the probe compared with NIR formulations and this appears entirely driven by the radioisotope and its chelator rather than the peptide compound of the RIP. (**d**) Radioisotope deposition enables similar quantification of thrombin generation. The difference in signal compared with a no thromboplastin control is plotted. %ID cc^−1^ is the amount of radioisotope injected per cubic centimetre of tissue as sampled through the lung of the mouse. Data are mean±s.d. of three biological replicates. All results are expressed as *P*<0.05, one-way ANOVA followed by Dunnett’s post-test. cps, counts per second.

The non-invasive imaging of a haemostatic process was achieved in a wounding model using a 28-gauge needle to create a small puncture wound in the hind leg of a mouse shortly after tail-vein injection of ATTO 680–PAR1–RIP (Fig. 6a). The signal detection arising from a region of interest defined at this site over time revealed an eightfold gain in signal over sham wounding where probe was injected with the same conditions. Signal intensity saturated within twenty minutes of wounding (Fig. 6b). Initial velocity of probe deposition in this model suggests ∼10^10^ molecules of thrombin are generated by wounding the saphenous vein of a mouse with a 28-gauge needle. The short half-life of the probe does not enable an accurate assessment of the length of time in which thrombin is acting during this wounding model, and further studies coupling pharmacokinetics with intravital microscopy are required. Unlike the Cy7 variant, ATT0 680–PAR1–RIP showed limited liver accumulation and was primarily cleared via the renal system. These data highlight the utility of RIP technology for real-time quantification of proteolytic events *in vivo* through non-invasive detection.

### Non-invasive imaging of PE using a PET/CT RIP

Encouraged by the ability of PAR1–RIP to accumulate in sufficiently high copy number for the non-invasive imaging of non-lethal PE, a variant of the peptide was created bearing diethylene triamine pentaacetic acid (DTPA)—a broad spectrum metal chelator—for nuclear imaging. DTPA–PAR1–RIP was labelled with the positron-emitting radioisotope ^68^Ga (t_1/2_ 68 min) for positron-emission tomography (PET) imaging. The dynamic capabilities of PET were used to image picomolar RIP dosing regimens in the PE model. Similar to that observed with Cy7–PAR1–RIP, the nuclear imaging variant accumulated in the lungs of the mice when co-administered with thromboplastin but not in untreated mice (Fig. 6c). RIP technology was used to deposit radioisotopes in high abundance as indicated by a fivefold gain in signal-to-noise and a per cent injected dose per cubic centimetre (%ID cc^−1^) exceeding 5% to the sites of protease activity. Probe accumulation ceased within the first 4 min of accumulation in the lung (Fig. 6d). The rate of probe deposition cannot quantify thrombin generated in this model, as the liver rapidly took up the metal-chelated probe similar to the Cy7 variant described previously. Nonetheless, non-invasive imaging of clot formation using a relevant imaging modality suggests the potential for clinical translation of our RIP technology.

## Discussion

To enable non-invasive real-time imaging of protease activity, we developed a scaffold differing significantly from past approaches and coupled biochemistry more directly to cell biology. Hairpin-structured activatable cell-penetrating peptides have been proposed to mediate intracellular delivery in response to metalloproteases^31^ and more recently thrombin^26^. Active site-dominated contacts limit the specificity of these probes and their protease-responsiveness is limited^32^. Unlike cell-penetrating peptides, our polymers have a defined localization mechanism involving formation of an α-helix on membrane interaction, lack a high density of cations and do not transpose into the cytoplasm. Our thrombin-specific probes exhibit 100-fold lower *K*_*M*_ values than comparable activatable cell-penetrating peptides^27^ that localize after activation. The lower *K*_*M*_ values in turn enable 100-fold lower dosing amounts, more rapid imaging times and lower background noise. These features are also significantly improved over commercially protease-targeted imaging agents that require 8–24 h delay from time of injection to imaging and are not documented to respond to thrombin. Similarly, approaches for PS-based imaging have been presented, but lacked a mechanism whereby the natural presentation of the phospholipid at low levels is accounted for and the probes have poor pharmacokinetic properties^33^. In another approach an IgG-based, platelet-targeted sensor was used to reveal thrombin gradients during clot formation. This sensor has specificity restricted to one cell type, since it is based and driven by an antibody directed against the platelet marker CD41 (ref. 34).

Preference for PS provided our technology an unexpected enhancement on the target cells and low non-specific accumulation. PS constitutes between 2 and 10% of total cellular phospholipid yet nearly all of this material is maintained in the inner leaflet of healthy cells^35^. Translocation of PS to the outer membrane is a hallmark of cell death, cancer, and activated platelets during blood clotting. Probes that detect PS have been investigated as stand-alone non-invasive imaging agents to measure disease severity and response to therapeutic intervention^36^,^37^. However, the large surface area of the vasculature presents a formidable obstacle for obtaining clear images of disease solely based on the presence of PS and improved probes are sought. RIPs bind cell membranes after proteolytic activation at sites of disease in live animals. In the absence of a proteolytic trigger, they have little affinity for cell membranes and, owing to their small size, are rapidly removed from circulation. Proteolysis unleashes membrane-binding activity of the RIP and this is more pronounced when the associated cells also present PS; a useful pairing for the detection of blood clots and other diseases. Coupling selective proteolysis and PS exposure in the RIP approach creates a useful platform for functional imaging using optical or nuclear modalities by enabling picomolar probe dose sizes, low background signal, real-time non-invasive measurement and signal tracking in sectioned tissues to resolve spatial heterogeneity of biochemical events. The algorithmic nature of AMP structure and function affords the potential for incorporating non-natural amino acids, therapeutic modalities, imaging agents or pharmacokinetic modifiers. The current peptides are not as water soluble as envisaged likely due to the length of peptides but our results suggest the potential for optimization. Improving water solubility and lengthening circulating half-life are tractable problems that could be achieved through pegylation or D-amino acid exchange. These and other improvements such as enhancement of restricted activity (for example, adding different polyanionic sequences to the protease target domain) may eventually facilitate RIP technology applications in non-invasive clinical diagnostics and prognostics.

## Methods

### Reagents

The lipids, egg L-α-PC, brain L-α-PS, L-α-phosphatidylethanolamine and dodecylphosphocholine were purchased from Avanti Polar Lipids, Inc. (Alabaster, AL). Polycarbonate filters for extruded lipid vesicle preparation using the LiposoFast system from Avestin, Inc. (Ottawa, Ontario, Canada). Other chemicals were of analytical grade and of the highest purity and were purchased from Sigma-Aldrich (St. Louis, MO). Wheat-germ agglutinin (WGA) conjugated with Oregon Green 488 was from Invitrogen (Carlsbad, CA). DRAQ7 was from BioStatus (Shepshed, UK). Procoagulant and anticoagulant proteases were from Haematologic Technologies (Essex Junction, VT).

### Liposome preparation

Liposomes were prepared by rehydration and extrusion. PS, PC or phosphatidylethanolamine were dissolved in chloroform at a desired molar ratio (80–125 μM) and the solvent removed under vacuum in a rotary evaporator for at one hour. The resulting lipid film was dried under stream nitrogen to remove trace solvent. The lipid film was then hydrated at room temperature (RT) in phosphate buffer (pH 7.4) with vortexing. Followed by three freeze-thaw cycles in a dry ice-ethanol bath. Large unilamellar vesicles were prepared by extrusion of the lipid suspension through 100-nm pore-size polycarbonate filters. Liposomes were used within 1 week of their preparation.

### Peptide synthesis and fluorophore labelling

Peptides were synthesized on an automatic peptide synthesizer by using standard protocols for fluorenylmethoxycarbonyl solid-phase synthesis. All peptides were at least 95% pure by high-performance liquid chromatography (HPLC) and further characterized through mass spectrometry.

### Tryptophan fluorescence spectroscopy

Emission spectra of the intrinsic fluorescence of tryptophan within the membrane-binding segment of the peptide were acquired using a Fluorolog-3 spectrofluorometer (HORIBA Jobin Yvon, Longjumeau, France). Vesicle suspensions were prepared as for solid-state NMR experiments above in the absence of peptide, except that the freeze-thaw cycles were omitted, yielding large multilamellar vesicles. Vesicles containing POPC/POPS (75:25) and POPE/POPS (75:25) were prepared at a concentration of 5 mg ml^−1^. From these suspensions, 150 μl was added to 0.85 ml PBS, and then peptide in solution (2 mg ml^−1^ in PBS) was added to produce a final peptide concentration of ∼0.01 mg ml^−1^. A peptide/lipid molar ratio of 1:40 was maintained. Tryptophan emission spectra of the lipid/peptide suspension were acquired by scanning from 310 to 450 nm using an excitation wavelength of 295 nm and a spectral bandwidth of 5 nm for both excitation and emission. A spectrum of the aqueous peptide was acquired at a peptide concentration of 0.1 mg ml^−1^ in the same buffer. All spectra are an average of three scans. The temperature was maintained at either 310 or 298 K by connecting the cuvette holder to an external water bath.

### Circular dichroism

Spectra were acquired on a Jasco J-810 spectrometer (Jasco, Tokyo, Japan) with samples maintained at 310 K. Spectra were recorded from 250 to 190 nm using a spectral bandwidth of 1 nm and a scan rate of 100 nm min^−1^. Samples were prepared as for the fluorescence experiments above, but with the lipid suspension undiluted. From the lipid suspension, 240 μl was added to a 1-mm cuvette and then 12 μl peptide solution (2 mg ml^−1^) was added and thoroughly mixed. Spectra were treated using Jasco spectra analysis software, where a spectrum of the peptide-free suspension was subtracted and means-movement smoothing with a convolution width of five points was applied.

### Peptide kinetics

Human clotting factors IIa (thrombin), VIIa, IXa, Xa, XIa, plasmin and aPC were obtained from Haematologic Technologies (Vermont, USA). Kinetics studies of probe digestion were measured by HPLC over the course of 1 h at 37 °C, using 10 nM of enzyme and 1 μM of PAR1–RIP in PBS (pH 7.4). Reactions were terminated with 20 μl of 1 M perchloric acid. Time-dependent digestion of PAR1–RIP was confirmed by mass spectrometry.

### Live-cell fluorescence microscopy

The human cancer cell lines were plated in 35-mm glass-bottom culture dishes (MatTeK) and allowed to grow in DMEM supplemented with 1% (vol/vol) GlutaMAX, 10% FBS and 1% penicillin–streptomycin until the cells were 75–90% confluent. Before imaging, the cells were washed with PBS and allowed to grow in serum-free DMEM for 18 h at 37 °C under 5% CO_2_. A11-AF488 was prepared using a previously reported protocol^38^. Serum-free media were removed, and the cells were washed twice with PBS before A11-AF488 in serum-free media was added to a final dye concentration of 100 nmol l^−1^ (approximate final volume was 2.3 ml). The culture dishes were returned to the incubator for 2 h, after which the plates were washed with PBS to remove any unbound antibody and 2 ml of phenol red-free, serum-free media was added for imaging. The cells were imaged within 10 min of removal from the incubator using a Nikon 6D high throughput epifluorescence microscope at the Nikon Imaging Center at the University of California at San Francisco/QB3.

### Fluorescence-activated cell sorting analysis and microscopy

Jurkat cells provided by UCSF Cell Culture Facility (clone E6-1, ATCC) were cultured in RPMI medium 1640 plus 10% (vol/vol) FBS to a density of 0.5–1 × 10^6^ cells per ml. The media was refreshed 1 day before assaying with RIPS. Cells were washed with Hanks’ balanced salt solution (HBSS) buffer three times, resuspended in HBSS at 0.5–1 × 10^6^ cells per ml, stained with 1 μM peptide in HBSS at RT for 10 min, washed three times with cold HBSS and analysed by flow cytometry at 530-nm emission for fluorescein-labelled peptides or at 675 nm for Cy5-labelled peptides. We collected 10,000 events from cells judged to be healthy by their forward and side scatter. Peptide association with HT-1080 cells was similarly quantified by flow cytometry after release from adherence with trypsin. For microscopic imaging, HT-1080 cells grown to 70% confluency were washed with HBSS three times, stained with 1.25 μM peptide and 1 μg ml^−1^ Hoechst 33258 (a nuclear stain), rinsed twice, trypsinized and replated on polylysine-coated dishes and, imaged for Cy5 content (excitation 625–645 nm; emission 665–695 nm) and Hoechst 33258 (excitation 375–385 nm; emission 420–460 nm).

### Animals

Adult wild-type C57BL/6J mice and *Foxn1*^*nu*^ from both genders, weighting 10–30 g were obtained from The Jackson Laboratory (Bar Harbor, ME). Experiments were in compliance to the UCSF Institutional Animal Care and Use Committee (IACUC) who approved all animal care and experimental procedures. The number of mice chosen to confirm our hypotheses was 128 and were derived from three mice groups: (A, *n*=44) controls where no procoagulant is injected, or a procoagulant may or may not be injected and tested with an uncleavable RIP; (B, *n*=50) RIP probe is injected over a time course following thrombus formation; and (C, *n*=34) RIP is injected over a time course in the presence of a specific inhibitor; nude mice, *n*=33; C57BL/6, *n*=95. An error threshold of 10% relative to the injection volume was maintained as adopted as sample exclusion criteria (mice not directly injected with <90% of initial volume were excluded from statistical analysis.

### Imaging arterial thrombosis in the mouse carotid artery

The ferric chloride (FeCl_3_)-induced vascular injury was performed as described elsewhere^39^. Briefly, Cy5–PAR1–RIP or its Cy5-uncleavable variant was administered by tail-vein injection as previously described for the pulmonary embolism model and a midline incision made through the skin from sternum to chin of anaesthetized C57BL/6J mice to expose the carotid artery. After exposure, a Whatman filter saturated in 4% FeCl_3_ solution was placed on the carotid artery for 3 min. Complete occlusion was observed within 20 min after contact with FeCl_3_ generating clots of ∼1 mm^3^ in size. Fluorescence signal was obtained from the damaged area and compared with that from the upstream healthy vessel to further access the signal-to-noise ratio. Fluorescence capture was performed using the IVIS imaging system with excitation of 640 nm and emission 700 nm.

### Thromboplastin-induced pulmonary embolism

We used a model similar to that described previously^30^. Thromboplastin (Sigma-Aldrich) used for these studies was from rabbit brain. Each vial of thromboplastin (3–4 mg) was resuspended in 4 ml saline and 100 μl of this solution was injected per mouse as below. The dose range examined had a 100% survival rate. As described previously female mice were used as preliminary studies revealed a sex difference in sensitivity to thromboplastin^40^. Briefly, anaesthesia was induced in *Foxn1*^*nu*^ or C57BL/6J subjects with 3–4% isoflurane having appropriate oxygen flow. Once animals reached deep anaesthesia, they were maintained at 2% isoflurane with continuous monitoring. With the animals under deep anaesthesia, thromboplastin (4 mg ml^−1^) isolated from rabbit brain (Sigma-Aldrich) was administered via tail-vein injection to reach sub-lethal doses in each mouse while triggering the formation of micro emboli. After 10 min from the initial injection, PAR1–RIP (10 μM kg^−1^) prepared in PBS (pH 7.4) containing polyethylene glycol 16,000 and no more than 15% dimethylsulphoxide (DMSO) was injected via tail vein to reach a final concentration of 500 picomoles in each mouse.

### Histology

Histological staining was conducted on lung tissue sections embedded with paraffin by the UCSF Tissue Core. Lung tissue sections were deparaffinized with xylene and alcohol, and were then subjected to standard haematoxylin and eosin staining. Fluorescent staining of the tissues was performed with standard protocols and the FITC-anti-platelet CD41 (WReg30-PE clone, eBioscience) solution was prepared as a 1:100 dilution. Briefly, frozen slides were rehydrated in PBS for 1 h with buffer exchange each 20 min. Excess PBS was removed from the samples, which were further overlaid with CAS-Block reagent and incubated at RT for 10 min. Excess blocking reagent was removed and the samples overlaid with 125 μl PBS (0.4% bovine serum albumin (BSA)) containing 5 μl of FITC CD41 and incubated overnight at −4 °C in a sealed container. After incubation the samples were washed twice in cold PBS for 10 min each. Next, the samples were overlaid by 125 μl of PBS (0.4% BSA) containing 0.2 μl 4,6-diamidino-2-phenylindole (DAPI; 10 mg ml^−1^), 5 μl WGA Texas Red (1 mg ml^−1^) and 1 μl of FITC CD41 and incubated at RT for 10 min. After washing three times in cold PBS, buffer was removed and samples overlaid with 35 μl Mowiol 4-88 (90%) mixed with ProLong Gold antifade (10%). A coverslip was added to the mount and the slide incubated at 37 °C for 10 min before imaging.

### NIR optical imaging

Nude mice for the optical studies (*n*=5 per treatment) were fed an alfalfa-free diet of Harlan Teklad Global 2018 to minimize background fluorescence. The mice were anaesthetized with 2% isoflurane. PAR1–RIP conjugated to ATT0680 or Cy7 were diluted in PBS containing 0.1% PEG 16,000 and no more than 15% DMSO and administered at a final concentration of 500 picomoles for each mouse. Peptide was injected via the tail vein of the mice at a volume 150 μl ten minutes after the injection of thromboplastin or 10 min before wounding. Images were collected in fluorescence mode on an IVIS spectrum using Living Image software. Region of interest measurements were made, the fluorescence emission images were normalized to reference images and the radiant efficiency was computed.

### Positron-emission tomography

^68^Ga for the radiolabeling reaction was obtained by the fractioned elution of a commercially available ^68^Ge/^68^Ga generator in 1 ml of 0.1 M HCl. The acidic elution solution was removed and the ^68^Ga was resuspended in a 25-mM sodium acetate solution (pH 4.0). The labelling of the DTPA-conjugated peptide with ^68^Ga was accomplished using standard chelation conditions. Briefly, 10 μM DTPA–PAR1–RIP in a 100 μl volume of 25 mM sodium acetate (pH 4.0) was added to an equal volume of ^68^Ga (10 mCi) and incubated at 37 °C for 60 min. Labelling efficiency was determined by radioactive thin-layer chromatography to be >99%. After labelling, the pH was adjusted to neutrality by the addition of 1.25 M sodium acetate and the samples were diluted with PBS for injection without any further purification. PET scans were performed on a PET/computed tomography (CT) scanner (Inveon, Siemens Healthcare, Malvern, PA). Mice (*n*=5 per treatment) fasted overnight and were injected intravenously with 150–200-μCi ^68^Ga-labelled DTPA–PAR1–RIP corresponding to a peptide mass of 200 picomoles of peptide per injection of 150 μl. PET images were acquired immediately after injection in a 1,200-s frame. CT images were acquired in 120 projections of continuous rotation to cover 220 degrees with an X-ray tube operating at 80 kVp, 0.5 mA and 175 ms exposure time. PET images were reconstructed using a manufacturer-provided ordered subsets expectation maximization algorithm resulting in a 128 × 128 × 159 matrix with a voxel size of 0.776 × 0.776 × 0.796 mm^3^. The data were analysed with the AMIDE and AMIRA software packages.

### Acute toxicology

To address the safety profile of this novel functional imaging platform, PAR1–RIP probe was administered in C57BL/6J mice at a concentration threefold higher than used for the imaging studies (150 picomoles, 100 μl). After 6, 24 and 72 h (*n*=3 per time point), mice were killed and blood was collected in BD Microtainer tubes and allowed to clot at RT before spinning for 10 min at 8,000 *g*. The serum was pipetted into clean tubes and frozen before shipment to IDEXX BioResearch (Sacramento, CA) for a comprehensive panel of hepatic and renal functions. All tests were performed using an Olympus 5400 analyser. Measurements of the following enzymes and electrolytes were provided: alkaline phosphatase, alanine aminotransferase, aspartate aminotransferase, creatine phosphokinase, total protein, direct bilirubin, indirect bilirubin, total bilirubin, globulin, blood urea nitrogen, creatinine, cholesterol, glucose, calcium, phosphorus, bicarbonate, chloride, potassium, sodium and haemolysis index.

### Haemolysis of human erythrocytes

The haemolytic activity of the Cy7–PAR1–RIP variants was measured on healthy human erythrocytes and compared with the Cy7-wild-type TempL as reported previously^41^. Briefly, aliquots of human erythrocyte suspension in PBS (pH 7.4) were incubated with serial dilutions of the peptides (dissolved in DMSO before use) for 1 h at 37 °C. The samples were then centrifuged for 5 min at 2,000 *g* and the release of haemoglobin was determined by monitoring the optical density of the supernatant at 540 nm. The experiments were performed in triplicate and complete haemolysis was determined using 1% Triton X-100. Haemolysis <5% indicated good haemocompatibility towards red blood cells. Informed consent was obtained from all human subjects and experiments performed in compliance to the protocol approved by the HUAP Research Ethics Committee, Brazil—ID number: 241.284. The usage of anti-inflammatory drugs, anticoagulants or hormones was adopted as sample exclusion criteria (donors under medical regimen that could affect normal haemostasis were not included in the study).

### Aggregation of washed human platelets

Washed platelets were prepared as described elsewhere^42^. Briefly, blood was collected from healthy donors using a BD Vacutainer Safety-Lok blood collection set with a 23-gauge needle. Platelet-rich plasma was isolated by centrifugation at 120 *g* for 15 min at RT and the platelet pellet was obtained by further centrifugation at 1,100 *g*. Platelets were washed with Hepes–Tyrode albumin buffer (10 mM HEPES, 0.2738 M NaCl, 5.4 mM KCl, 24 mM NaHCO_3_, 0.72 mM NaH_2_PO_4_, 2 mM MgCl_2_, 4 mM CaCl_2_, 10 mM dextrose and 0.7% BSA) at pH 6.5 in the absence of CaCl_2_ and resuspended in the complete buffer at pH 7.4. Cells from the washed platelet suspension were counted in a Hemavet 950 cell counter (154.3±14.4 × 10^3^ μl^−1^). Platelets were incubated with the probes, DMSO or argatroban (1.6 μM) for 2 min at 37 °C before addition of human thrombin (30 nM) and aggregation monitored by light transmission aggregometry using a Chrono-log aggregometer model 700. Informed consent was obtained from all human subjects and experiments performed in compliance to the protocol approved by the HUAP Research Ethics Committee, Brazil—ID number: 241.284. The usage of anti-inflammatory drugs, anticoagulants or hormones was adopted as sample exclusion criteria (donors under medical regimen that could affect normal haemostasis were not included in the study).

### Clot retraction assays

Clot retraction assays were performed as previously described^43^. Briefly, human blood was drawn from healthy donors and clotting was triggered in re-calcified (10 mM CaCl_2_) whole blood by bovine thrombin (2.5 U ml^−1^), in the presence of probes, DMSO or tirofiban (100 μM). Clotting and subsequent retraction proceeded in glass tubes kept at 37 °C in a water bath for 2 h. Clots were imaged and weighted using a Mettler Toledo precision balance NewClassic MS. Informed consent was obtained from all human subjects and experiments performed in compliance to the protocol approved by the HUAP Research Ethics Committee, Brazil—ID number: 241.284. The usage of anti-inflammatory drugs, anticoagulants or hormones was adopted as sample exclusion criteria (donors under medical regimen that could affect normal haemostasis were not included in the study).

### Statistical analysis

Toxicology results are presented as mean±s.e.m. Data were analysed using GraphPad Prism (GraphPad Software Inc., San Diego, CA) and statistical differences among experimental groups of animals were detected using one-way analysis of variance (ANOVA) followed by Dunnett’s post-test; *P*<0.05 was considered statistically significant.

## Additional information

**How to cite this article:** Page, M. J. *et al*. Non-invasive imaging and cellular tracking of pulmonary emboli by near-infrared fluorescence and positron-emission tomography. *Nat. Commun.* 6:8448 doi: 10.1038/ncomms9448 (2015).

## Supplementary Material

Supplementary Information Supplementary Figures 1-8, Supplementary Tables 1-2 and Supplementary References
