# Supplementary material for: Non-invasive imaging and cellular tracking of pulmonary emboli by near-infrared fluorescence and positron-emission tomography
Source: Nat Commun. 2015 Oct 1;6:8448. doi: 10.1038/ncomms9448 (PMC4593073; doi:10.1038/ncomms9448)
Supplement: Supplementary Information — Supplementary Figures 1-8, Supplementary Tables 1-2 and Supplementary References [file ncomms9448-s1.pdf]

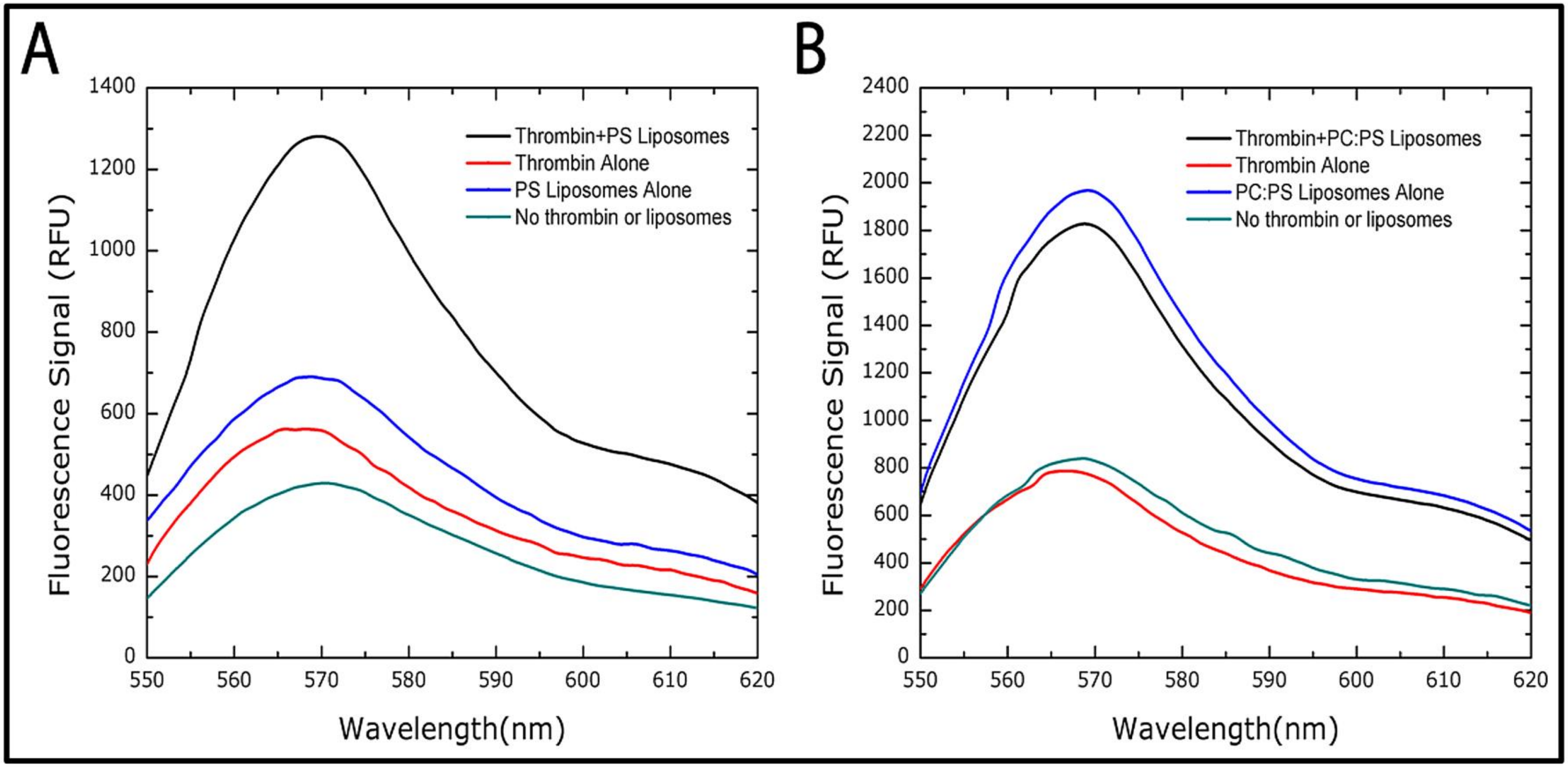

Supplementary Figure 1 – Binding of PAR1-RIP to (A) anionic liposomes consisting of phosphatidylserine and (B) zwitterionic liposomes composed of phosphatidylserine and phosphatidylcholine. The instrinsic fluorescence of the single tryptophan residue in the membrane interacting domain documents that thrombin activation was required for membrane insertion of the probe, independent of membrane curvature.

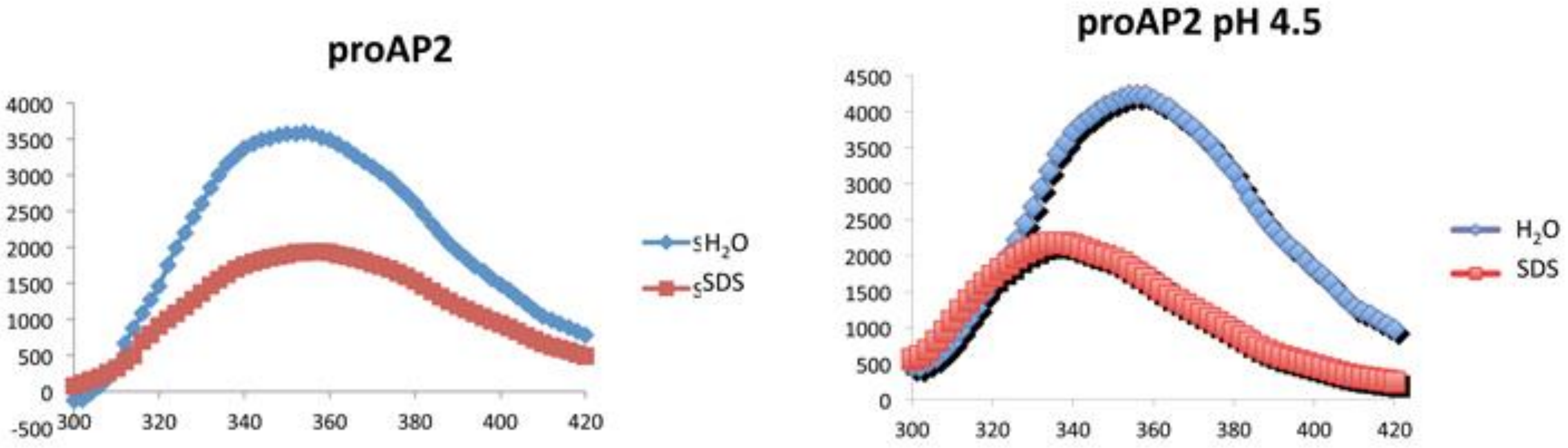

Supplementary Figure 2 – PAR2-RIP (proAP2) binds to SDS micelles at both neutral and acidic pH.

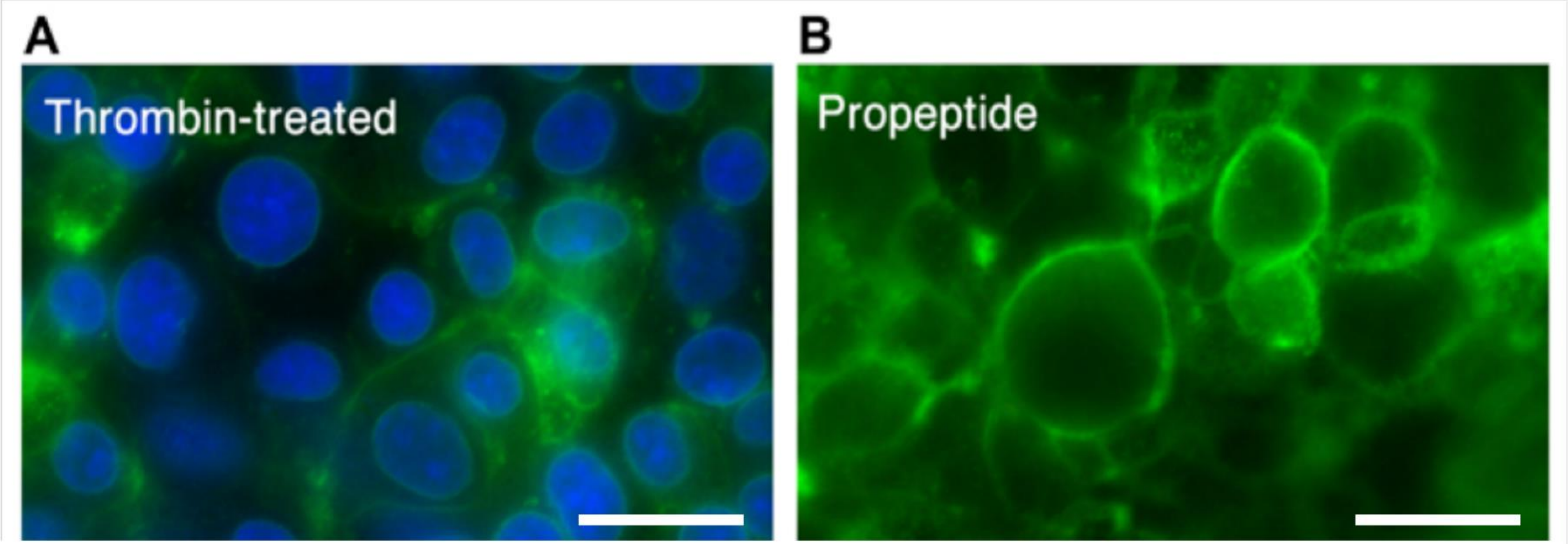

Supplementary Figure 3 – The cellular effects of the active PAR1-RIP peptide. High-doses (50 µM) of the cleaved PAR1-RIP lead to uptake of cell viability dyes including DRAQ7 (**a**), which does not occur with the propeptide (**b**) when incubated at same concentrations for extended periods of time (2 hrs). Scale bars, 20 µm.

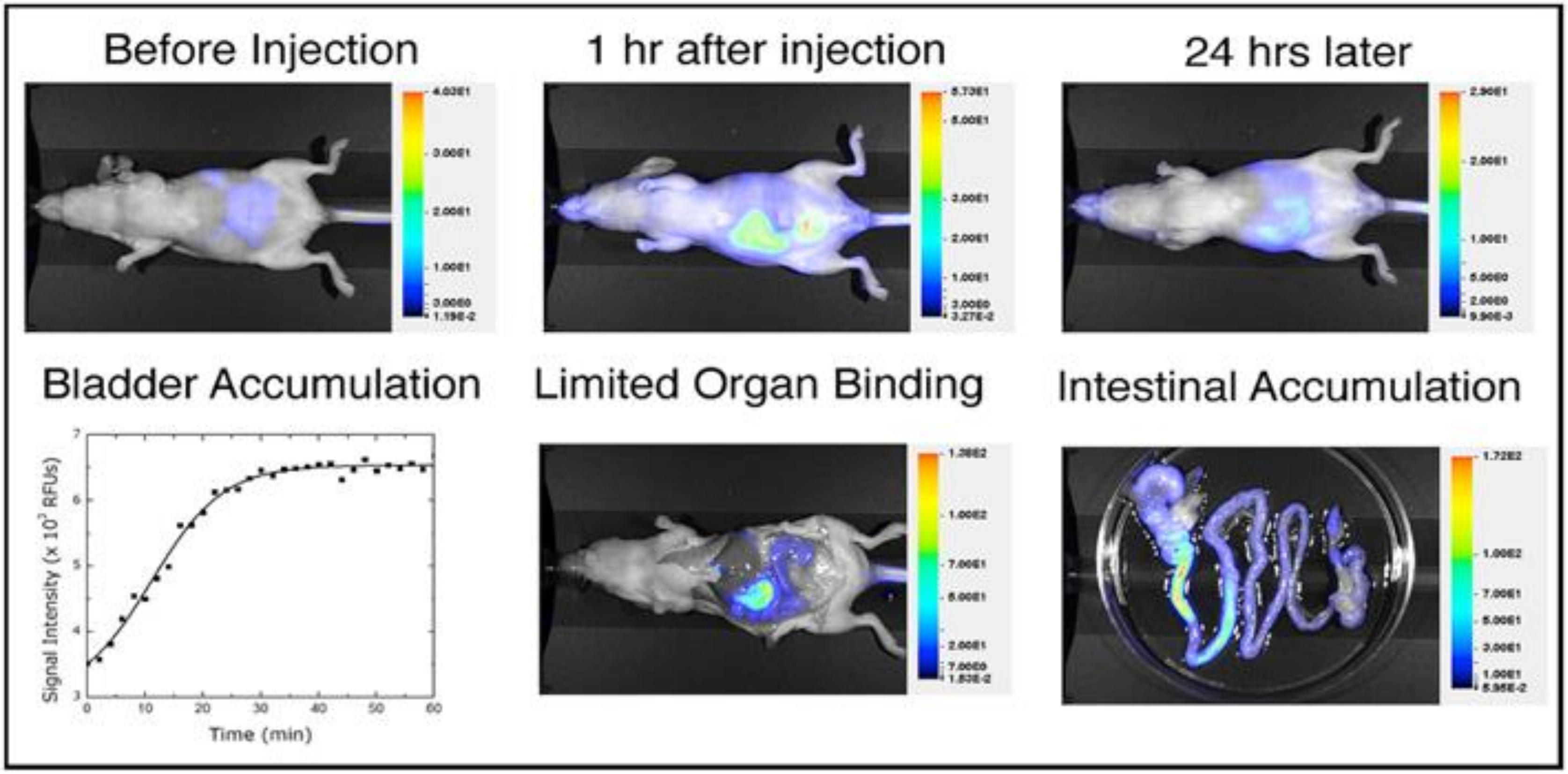

Supplementary Figure 4 - Clearance of PAR1-RIP. ATTO680-PAR1-RIP (500pmoles) was injected into normal healthy mice. Significant accumulation in the bladder was documented measuring the signal intensity of a region of interest containing the bladder. Probe accumulation in the bladder reached a plateau 30 minutes post-injection. In addition accumulation in the liver was observed followed by an increased signal in the intestines after 24h-post injection, suggestive of biliary elimination. Shown are representative images of pre, 1h and 24h post-injection of the probe.

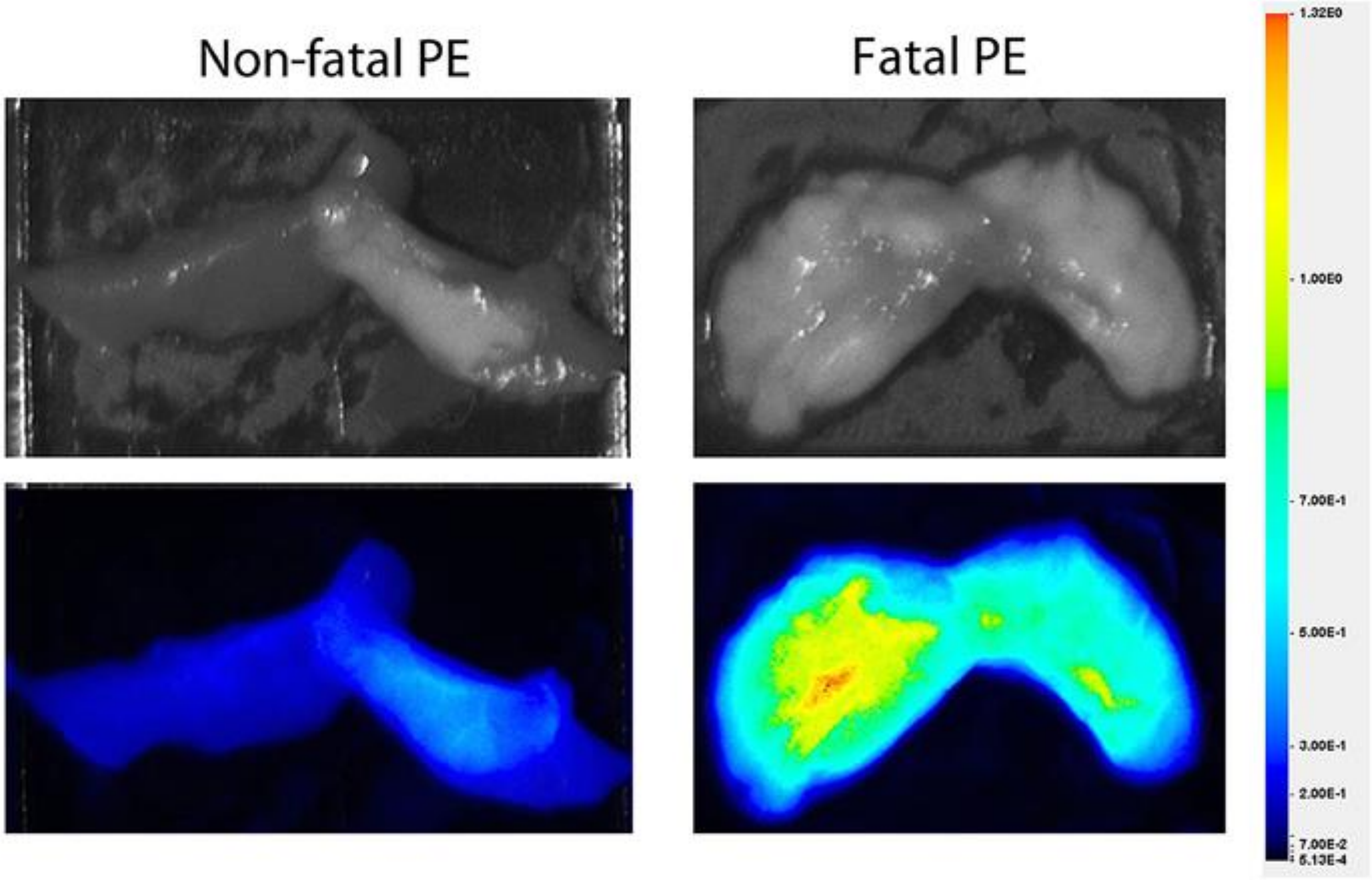

Supplementary Figure 5- The detection of Pulmonary emboli (PE) induced by thromboplastin using ATTO 680-PAR1-RIP (100 pmoles). For the non-fatal PE model, 4mg of thromboplastin was dissolved in 4 mL of saline (final concentration 1 mg/mL) and 100 μL were injected via tail vein into the mouse. After ten minutes, the probe was injected and the animals were sacrificed after additional ten minutes. The fatal PE model required a thromboplastin dose ten times greater than the non-fatal dose. The probe was injected ten minutes post-injection of thromboplastin and death typically occurred within 15 minutes.

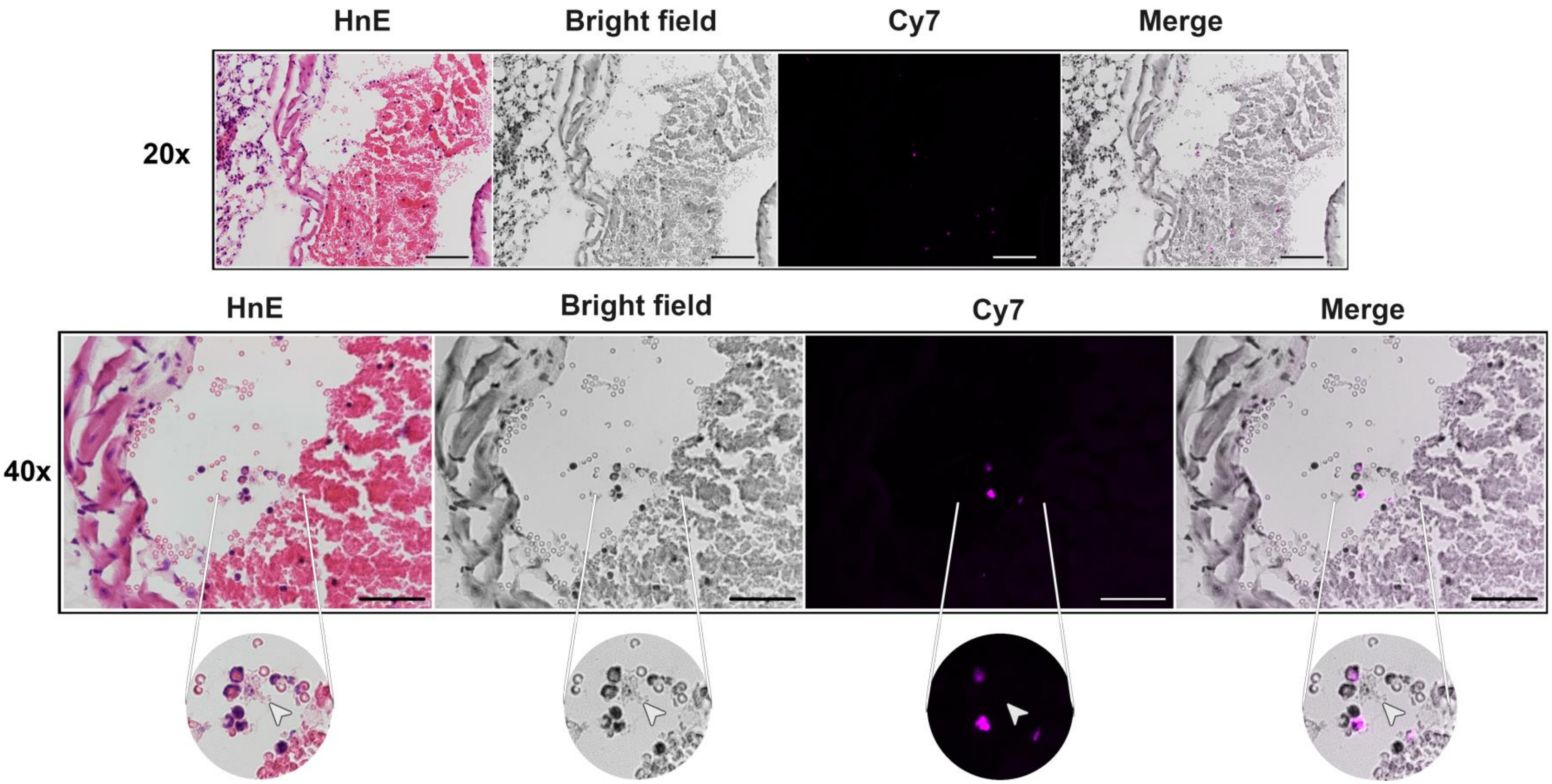

Supplementary Figure 6 - PAR1-RIP localizes in small clots found in non-occluded vessels. At 20x magnification (scale bars 100 µm) the Cy7 signal arising from PAR1-RIP (pseudo-colored pink) can be seen to localize at multiple small clots found in the non-occluded pulmonary vein filled with healthy blood cells. At higher magnification (40x, scale bars 50 µm) a platelet aggregate is seen in a fibrin network (arrow) also containing neutrophils, from which a strong Cy7 signal is observed, suggesting that the activation of PAR1-RIP at sites of thrombin activity leads to the rapid and active labeling of nearby cells.

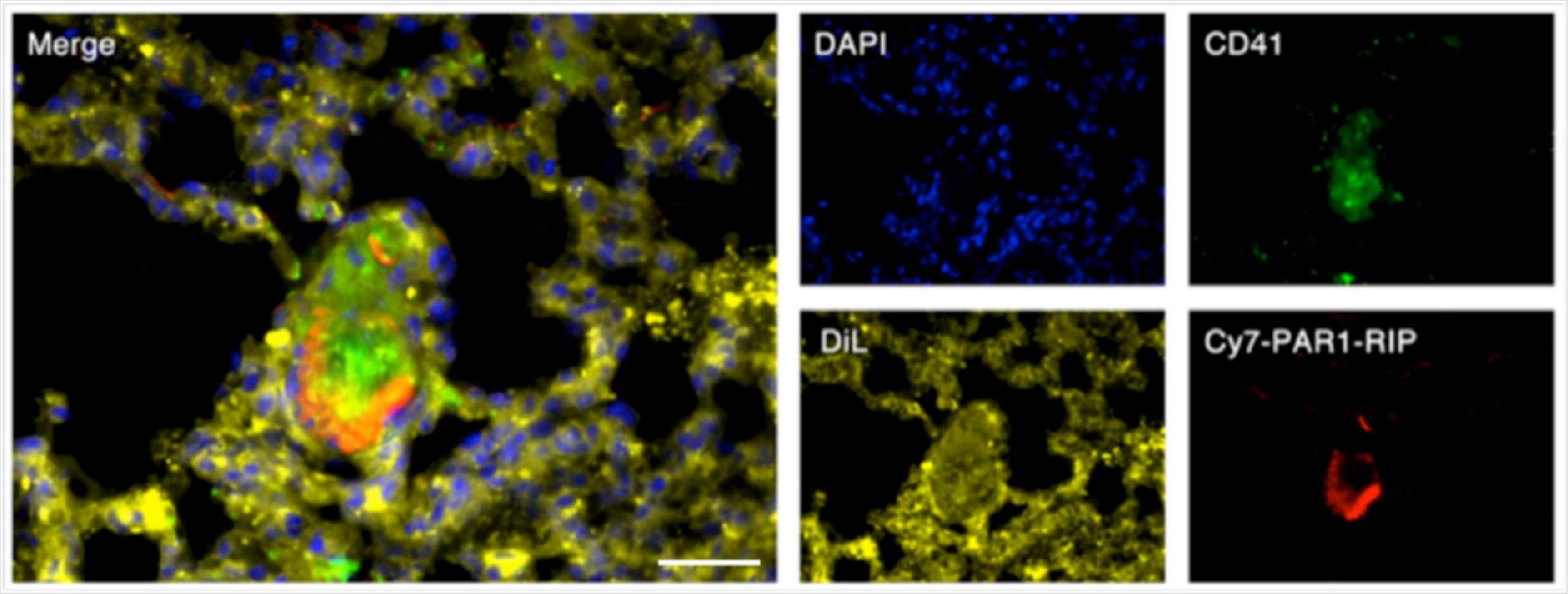

Supplementary Figure 7 - Fluorescence Microscopy of the lungs from mice injected with a non-fatal dose of thromboplastin. Cy7-PAR1-RIP (red) accumulates within the lumen of the vessel (scale bar, 25 µm) and on or near platelets (CD41, green). Cell nuclei revealed by DAPI (blue) and their membranes with the lipophilic dye DiL (yellow).

Structure	Name	Specific use referred in text
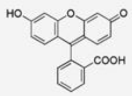	Fluorescein<br>(fluorescein-PAR1-RIP)	Imaging Thrombin Generation during *ex vivo* clot formation
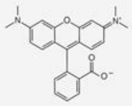	Tetramethylrhodamine	Screening the feasibility of PAR1-RIP to dye conjugation
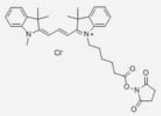	Cy3	Screening the feasibility of PAR1-RIP to dye conjugation
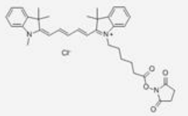	Cy5<br>(Cy5-PAR1-RIP)	Ferric Chloride-Induced Clot Formation in the carotid artery of mice
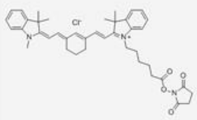	Cy7<br>(Cy7-PAR1-RIP)	Non-invasive Imaging of Pulmonary Emboli using Near-infrared Fluorescence<br>Hemolytic activity of PAR1-RIP imaging probes
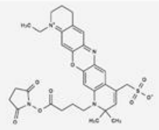	ATTO 680<br>(ATTO 680-PAR1-RIP)	Real-time Measurement of Thrombin Generation During Wounding<br>Qualitative analysis of biodistribution
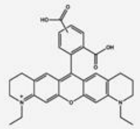	ATTO 565	Screening the feasibility of PAR1-RIP to dye conjugation

Supplementary Figure 8 – Structures of used fluorophores

#	Peptide	Wild-type Sequence	Optimizaed Sequence	Reference
**1**	Combi-2	FRWWHR	FRWWHKGSGR	Rezansoff et al. 2005
**2**	Jcpep7	KVFLGLK	KVWLGLKGSGR	Xiao et al. 2011
**3**	Myxinidin	GIHDILKYGKPS	GIHDILKWGKPSGSGR	Subramanian et al. 2009
**4**	Protonectin	ILGTILGLLKGL	ILGTILGLLKGLPR	Saidenberg et al. 2012
**5**	Combi-1	RRWWRF	RRWWRFGSGR	Rezansoff et al.2005
**6**	Jelliene-1	PFKISIHL	PWKLSLHLGSGR	Cabrera et al. 2014
**7**	Mellitin	GIGAVLKVLTTGLPALISWIKRKRQQ	LLPALISWIKRKRQQLVR	Werkmeister et al. 2002
**8**	Temporin-SHf	FFFLSRIF	FFWLSKIFGSGR	Abassi et al. 2010
**9**	Jelleine-2	TPFKISIHL	TPWKLSHLGSGR	Cabrera et al. 2014
**10**	Mastoparan B	LKLKSIVSWAKKVL	LKLKLIVSWAKKVLGSGR	Yang et al. 2013
**11**	Temporin L	FVQWFSKFLGRIL	FVQWFSKFLGKLLPR	Mangoni et al. 2011
**12**	Agelaia MP	INWLKLGKAIIDAL	INWLKLGKAIIDALGSGR	Saidenberg et al. 2012
**13**	IsCT	ILGTILGLLKGL	ILGKIWEGIKSLFAPR	Lee et al.2004
**14**	IsCT	ILGTILGLLKGL	ILGKIWEGIKLFGSGR	Lee et al.2004

Supplementary Table 1. List of corresponding peptides to the numbers found in **Figures 1b** and **1c**. The sequence of the original antimicrobial peptides used to construct each restricted interaction peptides is also provided for clearance.

	Control	PAR1-RIP 6 hs	PAR1-RIP 48 hs	PAR1-RIP 72hs	Units
Alanine Aminotransferase (ALT)	28.0 ± 4.5	29.7±1.4	26.3 ± 0.3	20.0±1.0	U/L
Aspartate Aminotransferase (AST)	68.7 ±14.5	61.7 ± 4.0	57.6 ± 6.3	53.7 ± 12.2	U/L
Creatinine	0.2 ± 0.0	0.2 ± 0.0	0.2 ± 0.0	0.2 ± 0.0	mg/dL
Creatinine Phosphokinase (CPK)	200.0 ± 44.2	144.7 ± 27.4	150.3 ± 38.0	156.3 ± 67.7	U/L
Albumin	2.7 ± 0.1	2.7 ± 0.1	2.6 ± 0.1	2.6 ± 0.0	g/dL
Total Bilirubin	0.1 ± 0.0	0.1 ± 0.0	0.12 ± 0.0	0.1 ± 0.0	mg/dL
Direct Bilirubin	0.0 ± 0.0	0.0 ± 0.0	0.1 ± 0.0	0.0 ± 0.0	mg/dL
Indirect Bilirubin	0.1 ± 0.0	0.1 ± 0.0	0.1 ± 0.0	0.1 ± 0.0	mg/dL
Blood Urea Nitrogen (BUN)	26.3 ± 2.2	27.0 ± 2.6	23.7 ± 1.4	26.7 ± 1.8	mg/dL
Alkaline phosphatase	111.7 ± 7.9	149.7 ± 12.8 *	120.7 ± 6.4	107.0 ± 7.8	U/L
Total Protein	4.6 ± 0.1	4.6 ± 0.1	4.6 ± 0.1	4.6 ± 0.1	g/dL
Globulin	1.9 ± 0.1	1.9 ± 0.0	2.0 ± 0.1	1.9 ± 0.1	g/dL
Cholesterol	74.0 ± 2.6	79 ± 0.6	84.3 ± 3.5 *	82.7 ± 1.7	mg/dL
Glucose	197.3 ± 28.9	238.7 ±2.9	203.0 ±3.5	268.0 ± 20.1	mg/dL
Phosphorus	7.1 ± 0.9	6.9 ± 0.2	6.8 ± 0.2	7.4 ± 0.2	mEq/ L
Bicarbonate	15.7 ± 0.3	13.3 ± 0.3 *	15.0 ± 0.0	18.3 ± 0.3 *	mEq/ L
Chloride	115.0 ± 3.2	116.0 ± 0.6	110.3 ± 0.8	110.3 ± 0.3	mEq/ L
Potassium	4.5 ± 0.3	4.7 ± 0.1	4.6 ± 0.1	4.9 ± 0.2	mEq/ L
Sodium	155.7 ± 1.8	152.3 ± 0.3	153.3 ± 0.9	150 ± 0.0 *	mEq/ L
Ratio Na/K	35.0 ± 2.6	32.0 ± 0.0	33.3 ± 0.1	30.3 ± 1.3	-
Calcium	9.2 ± 0.1	9.1 ± 0.1	9.0 ± 0.1	9.1 ± 0.2	mEq/ L
Hemolysis	2.0 ± 0.6	1.0 ± 0.6	1.3 ± 0.3	2.3 ± 0.7	%

Supplementary Table 2 - Investigation of the acute toxic effects caused by PAR1-RIP on liver and kidneys. Most of the 21 measured serum biochemical parameters were not different from controls at all time points, corroborating to the safety profile of PAR1-RIP.

Supplementary References

1. Rezansoff, A. J. et al. Interactions of the antimicrobial peptide Ac-FRWWHR-NH(2) with model membrane systems and bacterial cells. J. Pept. Res. Off. J. Am. Pept. Soc. **65,** 491–501 (2005).
2. Xiao, J., Zhang, H., Niu, L. & Wang, X. Efficient screening of a novel antimicrobial peptide from Jatropha curcas by cell membrane affinity chromatography. J. Agric. Food Chem. **59,** 1145–1151 (2011).
3. Subramanian, S., Ross, N. W. & MacKinnon, S. L. Myxinidin, a novel antimicrobial peptide from the epidermal mucus of hagfish, Myxine glutinosa L. Mar. Biotechnol. N. Y. N **11,** 748–757 (2009).
4. Baptista-Saidemberg, N. B. et al. Agelaia MP-I: a peptide isolated from the venom of the social wasp, Agelaia pallipes pallipes, enhances insulin secretion in mice pancreatic islets. Toxicon Off. J. Int. Soc. Toxinology **60,** 596–602 (2012).
5. Cabrera, M. P. dos S. et al. Combining experimental evidence and molecular dynamic simulations to understand the mechanism of action of the antimicrobial octapeptide jelleine-I. Biochemistry (Mosc.) **53,** 4857–4868 (2014).
6. Werkmeister, J. A., Hewish, D. R., Kirkpatrick, A. & Rivett, D. E. Sequence requirements for the activity of membrane-active peptides. J. Pept. Res. Off. J. Am. Pept. Soc. **60,** 232–238 (2002).
7. Abbassi, F. et al. Temporin-SHf, a new type of phe-rich and hydrophobic ultrashort antimicrobial peptide. J. Biol. Chem. **285,** 16880–16892 (2010).
8. Yang, M. J. et al. Enhancing antimicrobial activity of mastoparan-B by amino acid substitutions. J. Asia-Pac. Entomol. **16,** 349–355 (2013).
9. Mangoni, M. L. et al. Structure-activity relationship, conformational and biological studies of temporin L analogues. J. Med. Chem. **54,** 1298–1307 (2011).
10. Lee, K. et al. Antibiotic activity and structural analysis of the scorpion-derived antimicrobial peptide IsCT and its analogs. Biochem. Biophys. Res. Commun. **323,** 712–719 (2004).
